# Neck Circumference, a Novel Indicator for Hyperuricemia

**DOI:** 10.3389/fphys.2017.00965

**Published:** 2017-11-29

**Authors:** Jiajia Jiang, Jia Cui, Xinghua Yang, Anping Wang, Yiming Mu, Liguang Dong, Shuyu Wang, Herbert Gaisano, Jingtao Dou, Yan He

**Affiliations:** ^1^Department of Epidemiology and Biostatistics, School of Public Health, Capital Medical University, Beijing, China; ^2^Department of Endocrinology, Chinese PLA General Hospital, Beijing, China; ^3^Department of Endocrinology, Peking University Shougang Hospital, Beijing, China; ^4^Beijing Institute of Hypertension, Beijing, China; ^5^Departments of Medicine and Physiology, University of Toronto, Toronto, ON, Canada; ^6^Municipal Key Laboratory of Clinical Epidemiology, Beijing, China

**Keywords:** neck circumference, waist circumference, serum uric acid, hyperuricemia, association

## Abstract

**Background:** Waist circumference has been correlated with the risk of hyperuricemia. Whether neck circumference is also associated with hyperuricemia has not been assessed. This study aimed to investigate whether neck circumference is associated with hyperuricemia.

**Methods:** This study population from Beijing is part of the larger China-wide Risk Evaluation of Cancers in Chinese Diabetic Individuals: a lONgitudinal (REACTION) study. For this Beijing sub-center cross-sectional study, a total of 8971 subjects were recruited. Gender-specific multivariable-adjusted regression analyses were conducted to analyze the association of neck circumference and waist circumference with hyperuricemia and the association of neck circumference with serum uric acid levels in the non-hyperuricemia population.

**Results:** After adjusting for confounding variables, regression analyses showed that neck circumference was positively associated with hyperuricemia [OR, 2.61 (1.86–3.67) for males and 3.27 (2.53–4.22) for females] in both genders; further, neck circumference was also positively associated with serum uric acid levels in non-hyperuricemia subjects [b, 2.58 (1.76–3.39) for males and 4.27 (3.70–4.84) for females] in both genders. Additionally, we demonstrated that neck circumference was similar to waist circumference in terms of the strength of association (OR, 3.03 for waist circumference vs. 2.61 for neck circumference in males, and 3.50 vs. 3.27 for females) with hyperuricemia and the ability to predict hyperuricemia (AUC, 0.63 for waist circumference vs. 0.61 for neck circumference in males, and 0.66 vs. 0.66 in females).

**Conclusion:** Neck circumference is positively and independently associated with hyperuricemia in both genders and is also associated with serum uric acid levels in the non-hyperuricemia population.

## Introduction

Epidemiology studies from mainland China spanning 2000–2014 have shown that the prevalence of hyperuricemia is 13.7% in urban Chinese and 12.3% in rural Chinese individuals (Liu et al., 2015). Hyperuricemia causes gouty arthritis (Zamudio-Cuevas et al., 2015), kidney stones (Mirheydar et al., 2014) and chronic renal failure (Tsai et al., 2017), which significantly impact quality of life (Scire et al., 2013). Hyperuricemia has also been considered to be a risk factor for metabolic syndrome (Cibicková et al., 2017; Rubio-Guerra et al., 2017), diabetes mellitus (Wang et al., 2013), hypertension (Lyngdoh et al., 2012), stroke (Wu et al., 2017), chronic kidney disease (Ceriello et al., 2017) and cardiovascular disease (Qin et al., 2014; Amin et al., 2017; Moulin et al., 2017). Hyperuricemia is therefore a serious public health problem that should be detected early and treated. However, the predictors of hyperuricemia are not well-known.

Waist circumference, an indicator of abdominal obesity, has been reported to be associated with hyperuricemia in Chinese (Wang et al., 2013; Zhang et al., 2016), African Americans (McAdams-DeMarco et al., 2013) and Japanese individuals (Suma et al., 2014). The association of waist circumference and hyperuricemia was shown in a recent large study from China (Chen et al., 2017). The underlying mechanism linking waist circumference to hyperuricemia may be attributed to the excess free fatty acids released from the visceral fat, causing cellular fat accumulation and consequent lipotoxicity-mediated injury to multiple organs, which in turn would contribute to the metabolic disorder inflicted by uric acid on the kidney and liver (Weinberg, 2006; Yamada et al., 2016). Circulating free fatty acids have been postulated to emanate from the subcutaneous fat of the upper body (Martin and Jensen, 1991). Here, neck circumference was considered a predictive anthropometric measure of upper body fat distribution (Wang et al., 2015; Luo et al., 2017). Taken together, this finding serves as the rationale for our hypothesis that large neck circumference is associated with hyperuricemia. To the best of our knowledge, there has not been a study that has focused on assessing the association between neck circumference and hyperuricemia. A major aim of this study is therefore to examine whether neck circumference is associated with hyperuricemia and serum uric acid levels in non-hyperuricemia subjects. This study is also aimed at comparing the strength of association of neck circumference and waist circumference with hyperuricemia and their ability to detect hyperuricemia.

## Materials and methods

### Subjects

The data for this cross-sectional study were derived from a single city center, the Beijing sub-center, which is part of a much larger multi-center research study called the Risk Evaluation of Cancers in Chinese Diabetic Individuals: a lONgitudinal (REACTION) study (Ning, 2012). The REACTION project is a China-wide survey aimed at investigating the influence of metabolic diseases on cancer. The Beijing sub-center included 10 administrative regions, divided into 5 urban areas and 5 suburban areas. A total 10,276 participants aged 40 years or over were enrolled between April and October, 2015. After excluding subjects for any pathology or medical intervention that could alter the neck circumference, including neck malformation, prior neck surgery, thyromegaly, thyroid dysfunction and incomplete information for anthropometric parameters or laboratory examination, 8,971 subjects were finally included in our study. All subjects gave written informed consent in accordance with the Declaration of Helsinki. The protocol was approved by the Committee on Human Research of the Chinese People's Liberation Army General Hospital.

### Data collection

A standard questionnaire was completed by an in-person interview (between the survey interviewer and each study participant). Information on demographics, history of disease and corresponding medication use, smoking status and drinking status were collected during this process.

Physical examination was performed to obtain information on weight, height, neck circumference, waist circumference and blood pressure. Weight was measured to the nearest 0.5 kg by an electronic weight scale while participants wore light clothing (Beijing Jianmin); height was measured with a vertical height meter to the nearest 0.1 cm. The participants had to remove their shoes, hats, jackets, overcoats, and empty their pockets. Neck circumference was measured with a measuring tape along the inferior margin of the laryngeal prominence and perpendicular to the long axis of the neck, with the subject remaining standing and the head in the horizontal plane position. Waist circumference was measured at the horizontal plane between the inferior costal margin and the iliac crest on the mid-axillary line. All circumferences were recorded to within 0.1 cm. Blood pressure was measured three times at 1-min intervals using a mercury sphygmomanometer after at least a 5-min rest, and the mean value of the three readings was recorded.

The blood samples at fasting status were taken to determine fasting plasma glucose and uric acid levels and lipid profile. Participants were then subjected to a glucose load. Participants without a validated history of diabetes underwent a 75-g oral glucose tolerant test (OGTT), and participants with a history of diabetes underwent a 100-g carbohydrate diet test. Two hours after the OGTT or carbohydrate diet test, blood samples were taken again to determine the glucose tolerance of the subjects.

### Definitions

Body mass index (BMI) was calculated as weight (kilograms) divided by the square of the height (meters). Hypertension was defined as systolic blood pressure (SBP) ≥ 140 mm Hg and (or) diastolic blood pressure (DBP) ≥ 90 mm Hg or being on treatment for hypertension. Diabetes mellitus was diagnosed as fasting plasma glucose (FPG) ≥ 7.0 mmol/L or 2-h plasma glucose (2 hPG) ≥ 11.1 mmol/L, or being on treatment for diabetes. Hyperuricemia was defined as a serum uric acid level > 420 μmol/L in males and > 360 μmol/L in females, or subjects with gout or on hyperuricemia treatment. Current alcohol drinking status was classified into 3 categories: never drinking, occasional drinking (drinking less than once a week) and often drinking (drinking once or more a week). Current smoking status was classified into 3 categories: never smoked, occasional smoking (smoking less than one cigarette a day or seven cigarettes a week) and often smoking (smoking one or more cigarettes a day). Cardiovascular and cerebrovascular events included disease history of stroke and (or) myocardial infarction and (or) heart failure.

### Statistical analysis

All statistical analyses were performed with the SPSS software version 23 for Windows (SPSS Inc., Chicago, IL, USA, RRID:SCR_002865). Graphs were created using R version 3.3.1 (R development core team; available from http://www.r-project.org/, RRID:SCR_001905). The data were expressed as the means and standard deviations (SD) for continuous variables or numbers and percentages for categorical variables. The two-sample *t*-test and chi-square test were used to compare the differences in baseline characteristics for continuous and categorical variables, respectively. Correlations between variables and neck circumference or hyperuricemia were assessed by Pearson and Spearman correlation tests. The association of neck circumference and waist circumference with hyperuricemia was assessed by logistic regression analysis, while the association of neck circumference with serum uric acid levels was assessed by multivariable linear regression analysis. In the above regression analysis, the potential confounding factors were adjusted as indicated in the Results section. Receiver operating characteristic (ROC) cure analyses were used to identify hyperuricemia by neck circumference and waist circumference. All analyses were performed separately for gender. *P*-values less than 0.05 were considered statistically significant.

## Results

### Characteristics of the participants

In this cross-sectional study, 3,369 males and 5,604 females with a mean age of 60.0 ± 7.8 years were included. The mean serum uric acid was 341.5 ± 77.1 μmol/L (range from 112.0 to 755.2 μmol/L) and 281.5 ± 66.0 μmol/L (range from 106.8 to 664.2 μmol/L) for male and female participants, respectively. The prevalence of hyperuricemia in the male group (14.4%, *n* = 486) was 2.8% higher than that in the female group (11.6%, *n* = 651). Demographic information, indexes of body measurements and metabolic parameters of the subjects are shown in Table 1.

**Table 1:** Characteristic of participants categorized by gender and hyperuricemia status.

	**Total (*n* = 8,971)**	**Male (*****n*** = **3,369)**		**Female (*****n*** = **5,062)**	
		**Hyperuricemia (*n* = 486)**	**Non-hyperuricemia (*n* = 2,883)**	**Hyperuricemia (*n* = 651)**	**Non-hyperuricemia (*n* = 4,951)**
Age (y)^*^^#^	60.0 ± 7.8	60.9 ± 8.4	61.8 ± 7.9	60.7 ± 7.8	58.8 ± 7.5
Body mass index (kg/m^2^)^*^^#^	25.5 ± 3.5	26.7 ± 3.1	25.4 ± 3.2	27.5 ± 3.9	25.1 ± 3.6
Waist circumference (cm)^*^^#^	86.1 ± 9.5	93.2 ± 8.3	89.1 ± 8.6	88.7 ± 9.4	83.3 ± 9.2
Neck circumference (cm)^*^^#^	35.5 ± 3.3	39.1 ± 2.6	38.0 ± 2.6	35.1 ± 2.5	33.7 ± 2.4
Fasting glucose level (mmol/L)^*^^#^	5.9 ± 1.7	5.9 ± 1.5	6.1 ± 1.9	5.9 ± 1.3	5.7 ± 1.7
Glucose tolerance level (mmol/L)^#^	9.6 ± 3.8	9.4 ± 3.5	9.4 ± 4.2	9.8 ± 3.4	8.7 ± 3.7
Glycated hemoglobin (%)	6.1 ± 1.0	6.1 ± 1.0	6.1 ± 1.1	6.2 ± 1.1	6.1 ± 1.0
Systolic blood pressure (mm Hg)^#^	130.4 ± 17.0	134.2 ± 17.1	133.2 ± 16.9	131.9 ± 16.4	128.2 ± 16.8
Diastolic blood pressure (mm Hg)^*^	76.9 ± 9.9	79.8 ± 11.0	78.3 ± 10.2	76.5 ± 9.7	75.8 ± 9.5
Total cholesterol (mmol/L)	4.9 ± 1.7	4.7 ± 1.0	4.6 ± 2.2	5.1 ± 1.0	5.1 ± 1.4
Triglycerides (mmol/L)^*^^#^	1.6 ± 1.1	2.1 ± 1.6	1.5 ± 1.0	2.0 ± 1.3	1.6 ± 1.1
High density lipoprotein (mmol/L)^*^	1.5 ± 1.8	1.2 ± 0.3	1.3 ± 0.3	1.5 ± 2.8	1.6 ± 2.2
Low density lipoprotein cholesterol (mmol/L)	3.1 ± 1.4	3.1 ± 2.4	2.9 ± 1.4	3.2 ± 0.9	3.2 ± 1.3
Alanine transaminase(u/L)^*^^#^	20.7 ± 14.3	24.5 ± 15.6	20.8 ± 12.0	23.9 ± 17.4	19.9 ± 14.7
Aspartate transaminase(u/L)^*^	21.5 ± 28.6	23.5 ± 26.2	20.7 ± 18.4	22.3 ± 10.9	21.6 ± 34.6
γ-glutamyltranspeptidase(u/L)^*^^#^	27.7 ± 32.0	46.9 ± 71.7	31.4 ± 33.8	29.9 ± 23.5	23.4 ± 23.5
Creatinine (umol/L)^*^^#^	70.1 ± 16.6	90.7 ± 22.6	80.3 ± 13.6	70.0 ± 17.9	62.1 ± 11.4
Serum uric acid (mmol/L)^*^^#^	304.0 ± 76.1	473.6 ± 51.9	319.2 ± 55.3	403.8 ± 39.8	265.4 ± 49.9
Diabetes, *n* (%)^#^	2,554 (28.5)	155 (31.9)	954 (33.1)	242 (37.2)	1,203 (24.3)
Hypertension, *n* (%)^*^^#^	4,354 (48.5)	316 (65.0)	1,571 (54.5)	376 (57.8)	2,092 (42.2)
Cardiovascular and cerebrovascular events, *n* (%)^#^	447 (5.0)	31 (6.4)	197 (6.8)	40 (6.1)	179 (3.6)
**CURRENT SMOKING STATUS**, ***n*** **(%)**					
Never	7,335 (81.8)	284 (58.4)	1,590 (55.2)	632 (97.1)	4,829 (97.5)
Occasional	153 (1.7)	16 (3.3)	102 (3.5)	6 (0.9)	29 (0.6)
Often	1,483 (16.5)	186 (38.3)	1,191 (41.3)	13 (2.0)	93 (1.9)
**CURRENT DRINKING STATUS**, ***n*** (%)^*^					
Never	6,430 (71.7)	174 (35.8)	1,165 (40.4)	590 (90.6)	4,501 (90.9)
Occasional	1,345 (15.0)	127 (26.1)	813 (28.2)	44 (6.8)	361 (7.3)
Often	1,196 (13.3)	185 (38.1)	905 (31.4)	17 (2.6)	89 (1.8)

* *p < 0.05, comparison of hyperuricemia group to non-hyperuricemia group in male*.

# *p < 0.05, comparison of hyperuricemia group to non-hyperuricemia group in female*.

*Glucose tolerance level, blood glucose level for 2 h after the oral glucose tolerant test or carbohydrate diet test*.

### Neck circumference was associated with hyperuricemia

To precisely assess the association between neck circumference and hyperuricemia, we first determined the confounding factors between them. We identified the confounding factors as those that are both correlated with neck circumference and hyperuricemia. The results demonstrated that age, hypertension, diabetes, cardiovascular and cerebrovascular events, triglycerides (TG) and high-density lipoprotein (HDL) met the criteria for confounding factors (Table 2). Since current smoking and alcohol drinking status had been considered as confounding factors in previous studies (Zhang et al., 2016; Chen et al., 2017), we considered them to be additional confounding factors. We adjusted for these factors in the association analysis.

**Table 2:** Correlation of variables with neck circumference and hyperuricemia stratified by gender.

	**Male** ***(n*** = **3,369)**				**Female (*****n*** = **5,062)**			
	**Neck circumference**		**Hyperuricemia (*****n*** = **486)**		**Neck circumference**		**Hyperuricemia (*****n*** = **651)**	
	***R***	***P*-Value**	***r***	***P*-Value**	***r***	***P*-Value**	***r***	***P*-Value**
Age	−0.09	< 0.001	−0.04	0.01	0.05	< 0.001	0.08	< 0.001
Fasting glucose level	0.11	< 0.001	0.01	0.60	0.19	< 0.001	0.09	< 0.001
Glucose tolerance level	0.12	< 0.001	0.03	0.07	0.21	< 0.001	0.14	< 0.001
Glycated hemoglobin	0.01	0.52	0.02	0.21	0.03	0.036	0.02	0.19
Systolic blood pressure	0.16	< 0.001	0.02	0.17	0.23	< 0.001	0.07	< 0.001
Diastolic blood pressure	0.15	< 0.001	0.05	0.01	0.16	< 0.001	0.03	0.02
Total cholesterol	0.01	0.47	0.06	< 0.001	−0.03	0.03	0.004	0.74
Triglycerides	0.18	< 0.001	0.19	< 0.001	0.18	< 0.001	0.17	< 0.001
High-density lipoprotein	−0.31	< 0.001	−0.10	< 0.001	−0.05	0.001	−0.15	< 0.001
Low-density lipoprotein cholesterol	0.01	0.52	0.03	0.05	0.02	0.11	0.01	0.47
Alanine transaminase	0.16	< 0.001	0.10	< 0.001	0.15	< 0.001	0.11	< 0.001
Aspartate transaminase	−0.02	0.22	0.08	< 0.001	0.01	0.49	0.06	< 0.001
γ-glutamyltranspeptidase	0.08	< 0.001	0.16	< 0.001	0.12	< 0.001	0.17	< 0.001
Creatinine	0.08	< 0.001	0.20	< 0.001	0.07	< 0.001	0.19	< 0.001
Diabetes	0.12	< 0.001	−0.01	0.60	0.18	< 0.001	0.09	< 0.001
Hypertension	0.22	< 0.001	0.08	< 0.001	0.22	< 0.001	0.10	< 0.001
Cardiovascular and cerebrovascular events	0.05	0.002	−0.01	0.71	0.06	< 0.001	0.04	0.002
Current smoking status	0.02	0.24	0.02	0.18	0.04	0.002	0.01	0.49
Current drinking status	0.04	0.011	0.05	0.007	0.03	0.024	0.004	0.77

*Glucose tolerance level, blood glucose level for 2 h after the oral glucose tolerant test or carbohydrate diet test*.

To analyze the influence of neck circumference on hyperuricemia, we divided the subjects into 4 groups based on the quartiles of neck circumference. After adjusting for all of the confounding factors, the risk for male subjects in the second, third and fourth quartile for neck circumference being inflicted with hyperuricemia was 1.77-, 1.81-, and 2.61-fold the risk of males in the first quartile, respectively (Table 3). This finding indicated that the corresponding risk for the prevalence of hyperuricemia increased 0.77-, 0.81-, and 1.61-fold. For waist circumference, the corresponding risk was 1.83-, 2.10-, and 3.03-fold the risk of males in the first quartile, respectively (Table 3). The results of female subjects (Table 4) were similar to those of males for both neck circumference and waist circumference (Table 3).

**Table 3:** Association of body adiposity measures with hyperuricemia in male subjects.

	***n***	**Events, *n* (%)**	**Model I**		**Model II**		**Model III**	
			***P***	**OR(95%CI)**	***P***	**OR(95%CI)**	***P***	**OR(95%CI)**
**NECK CIRCUMFERENCE**								
Q1 (≤36.0)	880	68 (7.7)		Ref.		Ref.		Ref.
Q2 (36.1–38.0)	1,001	144 (14.4)	<0.001	2.01 (1.48–2.72)	<0.001	2.00 (1.48–2.72)	<0.001	1.77 (1.29–2.43)
Q3 (38.1–39.0)	898	142 (15.8)	<0.001	2.24 (1.65–3.04)	<0.001	2.20 (1.62–2.98)	<0.001	1.81 (1.31–2.50)
Q4 (≥39.1)	590	132 (22.4)	<0.001	3.44 (2.51–4.71)	<0.001	3.36 (2.45–4.61)	<0.001	2.61 (1.86–3.67)
**WAIST CIRCUMFERENCE**								
Q1 (≤83.9)	765	49 (6.4)		Ref.		Ref.		Ref.
Q2 (84.0–89.9)	895	116 (13.0)	<0.001	2.18 (1.54–3.09)	<0.001	2.15 (1.52–3.05)	0.001	1.83 (1.28–2.62)
Q3 (90.0–94.9)	802	121 (15.1)	<0.001	2.60 (1.83–3.68)	<0.001	2.58 (1.82–3.66)	<0.001	2.10 (1.47–3.02)
Q4 (≥95.0)	907	200 (22.1)	<0.001	4.13 (2.97–5.75)	<0.001	4.07 (2.93–5.66)	<0.001	3.03 (2.13–4.31)

*OR, odd ratio; CI, confidence interval; Ref, reference. Model I, unadjusted; Model II, Model I+ adjusted for age, current smoking status and current drinking status. Model III, Model II+ adjusted for Triglycerides, High-density lipoprotein, hypertension, diabetes, cardiovascular and cerebrovascular events*.

**Table 4:** Association of body adiposity measures with hyperuricemia in female subjects.

	***n***	**Events, *n* (%)**	**Model I**		**Model II**		**Model III**	
			***P***	**OR (95%CI)**	***P***	**OR (95%CI)**	***P***	**OR (95%CI)**
**NECK CIRCUMFERENCE**								
Q1 (≤32.0)	1,639	94 (5.7)		Ref.		Ref.		Ref.
Q2 (32.1–34.0)	998	79 (7.9)	0.028	1.42 (1.04–1.93)	0.033	1.40 (1.03–1.91)	0.127	1.28 (0.93–1.75)
Q3 (34.1–35.0)	1,603	202 (12.6)	<0.001	2.37 (1.82–3.06)	<0.001	2.33 (1.81–3.01)	<0.001	1.99 (1.53–2.58)
Q4 (≥35.1)	1,362	276 (20.3)	<0.001	4.18 (3.27–5.35)	<0.001	4.07 (3.18–5.22)	<0.001	3.27 (2.53–4.22)
**WAIST CIRCUMFERENCE**								
Q1 (≤76.9)	1,199	57 (4.7)		Ref.		Ref.		Ref.
Q2 (77.0–82.9)	1,317	112 (8.5)	<0.001	1.87 (1.34–2.59)	<0.001	1.84 (1.32–2.56)	0.004	1.63 (1.17–2.27)
Q3 (83.0–89.9)	1,621	196 (12.1)	<0.001	2.76 (2.04–3.75)	<0.001	2.63 (1.94–3.58)	<0.001	2.20 (1.61–3.01)
Q4 (≥90.0)	1,465	286 (19.5)	<0.001	4.87 (3.62–6.54)	<0.001	4.53 (3.37–6.11)	<0.001	3.50 (2.58–4.77)

*OR, odd ratio; CI, confidence interval; Ref., reference. Model I, unadjusted; Model II, Model I+ adjusted for age, current smoking status and current drinking status Model III, Model II+ adjusted for Triglycerides, High-density lipoprotein, hypertension, diabetes, cardiovascular and cerebrovascular events*.

**Figure 1:**
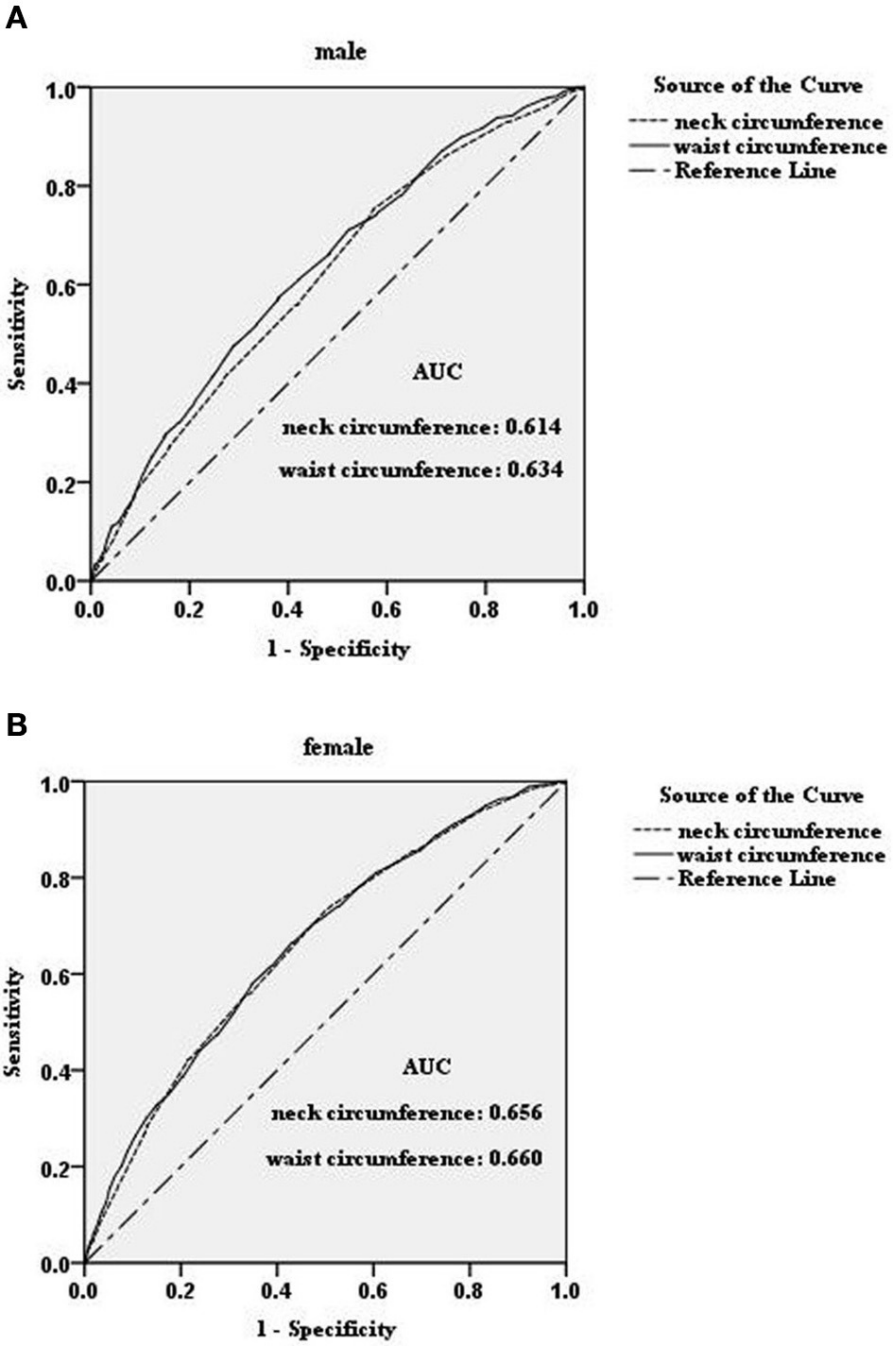
ROC-curves for neck circumference and waist circumference for distinguishing hyperuricemia in male **(A)** and female **(B)** subjects in the study population. AUC, area under the curve.

To compare the diagnostic value of neck circumference and waist circumference in identifying the presence of hyperuricemia, we performed a ROC analysis. The strengths of neck circumference and waist circumference in identifying hyperuricemia were very close in both genders, with the area under the curve (AUC) analysis as 0.61 vs. 0.63 in male subjects, respectively, and 0.66 vs. 0.66 in female subjects, respectively (Figure 1).

### Neck circumference was associated with serum uric acid levels in non-hyperuricemia subjects

**Figure 2:**
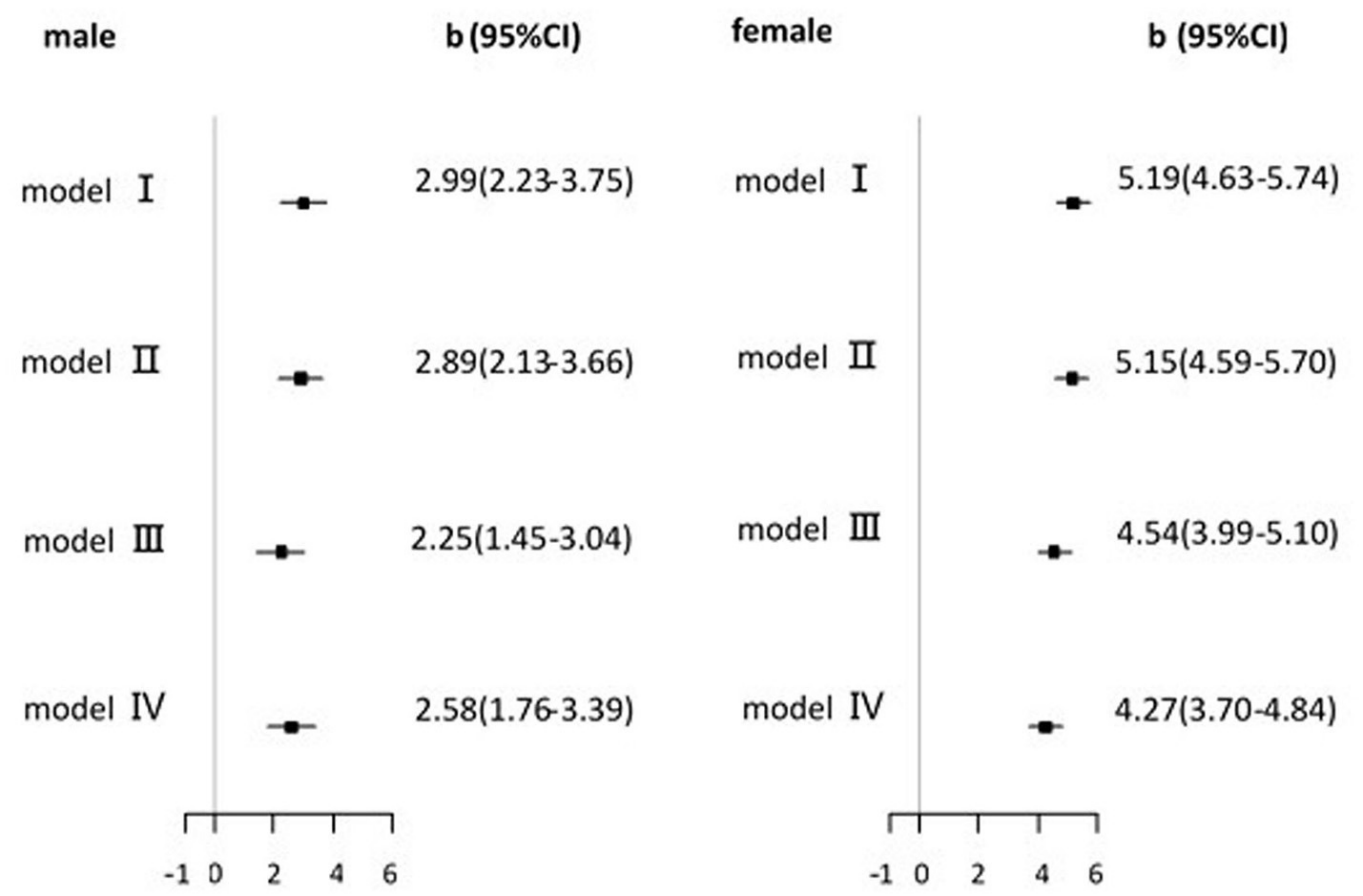
Association of neck circumference with serum uric acid level in non-hyperuricemia participants. b (regression coefficients) and 95% CI(confidence interval) from multivariable linear regression analysis. model I, unadjusted; model II, model I+ adjusted for age, current smoking status and current drinking status; model III, model II+ adjusted for TG and HDL; model IV, model III+ adjusted for hypertension, diabetes, cardiovascular and cerebrovascular events.

To examine whether neck circumference was also associated with the process of uric acid metabolism, we assessed the association between neck circumference and serum uric acid levels in the non-hyperuricemia subjects, which included 2,883 males and 4,951 females; the corresponding mean uric acid was 319.2 ± 55.3 μmol/L (range from 112.0 to 419.9 μmol/L) and 265.4 ± 49.9 μmol/L (range from 106.8 to 359.9 μmol/L), respectively. The characteristics of the non-hyperuricemia subjects are shown in Table 1. After adjusting for all confounding factors as indicated above, the neck circumference was positively associated with serum uric acid levels (*P* < 0.001). The strength of association was 2.58 (1.76–3.39) and 4.27 (3.70–4.84) for males and females, respectively (Figure 2). These results indicate that the neck circumference increased 1 cm each, and the plasma uric acid concentration increased 2.58 and 4.27 μmol/L in male and female subjects, respectively.

## Discussion

In this study, we demonstrated that neck circumference was significantly associated with hyperuricemia and that neck circumference was also positively associated with serum uric acid levels in non-hyperuricemia subjects. Additionally, these associations were independent of metabolic status and cardio-metabolic disease. To the best of our knowledge, this study is the first to demonstrate that neck circumference is correlated with hyperuricemia.

Both epidemiologic and clinical evidence indicated a close interrelation between hyperuricemia and obesity (McAdams-DeMarco et al., 2013; Shi et al., 2013; Wang et al., 2013; Suma et al., 2014). Waist circumference, an indicator of abdominal obesity, had already been determined to be associated with hyperuricemia (McAdams-DeMarco et al., 2013; Wang et al., 2013; Suma et al., 2014; Zhang et al., 2016). Large neck circumference, a marker of upper body adiposity (Wang et al., 2015; Luo et al., 2017), is similar to large waist circumference in its association with metabolic syndrome and cardiovascular disease (Hingorjo et al., 2016; Assyov et al., 2017; Luo et al., 2017). However, there has not been a study to assess the association between hyperuricemia and neck circumference. In this study, we demonstrated that neck circumference was similar to waist circumference in terms of the strength of association (OR, 3.03 for waist circumference vs. 2.61 for neck circumference in males and 3.50 vs. 3.27 for females) with hyperuricemia and their similar ability to predict hyperuricemia (AUCs of 0.63 for waist circumference vs. 0.61 for neck circumference in males, and corresponding AUCs of 0.66 vs. 0.66, respectively, for females). In this study, we also found that neck circumference was associated with the plasma levels of uric acid in non-hyperuricemia subjects. This finding indicated that neck circumference was not only a marker of hyperuricemia but was also associated with the process of uric acid metabolism.

We found that the strength of association between hyperuricemia/serum uric acid levels and neck circumference in females was stronger than that in males. This finding was similar to the result from Luo's study (Luo et al., 2017). The underlying mechanisms might be attributed to the different lipid metabolism and fat distribution between the two genders, which might be due in part to differences in sex hormones (Gu et al., 2017; Luo et al., 2017; Vashishta et al., 2017).

Visceral fat has been associated with hyperuricemia in a manner independent of the total fat area, total subcutaneous fat area, and abdominal subcutaneous fat area (Yamada et al., 2016). This finding, taken with the neck circumference as being positively associated with visceral obesity (Wang et al., 2015), led us to infer that visceral fat, but not total or subcutaneous fat, may mediate the linkage between neck circumference and hyperuricemia. This postulate is supported by our findings, which showed that after adjusting for triglycerides, a non-specific indicator of fat distribution, neck circumference was still significantly associated with hyperuricemia [OR, 2.61 (1.86–3.67)], although the strength of association was slightly decreased. Ectopic distribution of fat in or around the visceral organs, such as the liver and kidney, may cause fat infiltration and dysfunction of these organs. However, it is not clear how fat accumulation in these organs contribute to increased uric acid production in the liver or reduced excretion by the kidney, which are mechanisms that need to be explored. This finding emphasizes a limitation of this study, which is that we did not examine the visceral fat, especially with respect to hepatic and renal fat accumulation, which, if concurrently present with the high neck circumference, would confer upon the latter a possible mechanistic explanation for the association with hyperuricemia.

Hyperuricemia was reported as an independent and important risk factor not only for gout and hyperlipidemia (Hikita et al., 2007) but also for hypertension, diabetes (Miyagami et al., 2017) and cardiovascular morbidity and mortality rates (Grassi et al., 2013). There is therefore urgency in elucidating the more clinically apparent markers for hyperuricemia, in part as predictors of these diseases. Waist circumference measurements may not be suitable for a number of clinical situations, such as for patients on bed rest or who are pregnant or who are in a number of disease states affecting waist circumference, such as ascites and abdominal tumors. Furthermore, methods for measuring waist circumference have not been clinically standardized (Wang et al., 2003; Willis et al., 2007; Bernritter et al., 2011). Thus, the utilization of waist circumference has been restricted, making the uniform and accurate measurement of neck circumference a better and more convenient clinical alternative.

## Conclusion

Our findings showed neck circumference to be positively and independently associated with serum uric acid levels and hyperuricemia in both genders. Neck circumference could be used as a predictive indicator to trigger the screening for and treatment of hyperuricemia.

## Nomenclature

### Resource identification initiative

The SPSS software version 23 for Windows (SPSS Inc., Chicago, IL, USA, RRID:SCR_002865).

R version 3.3.1 (R development core team; available from http://www.r-project.org/, RRID:SCR_001905).

## Author contributions

YH and JD conceived the study. AW, YM, LD collected population data. SW and HG performed laboratory assays. XY and JC performed statistical analyses. JJ wrote and revised the manuscript. JJ and JC interpreted the data and contributed equally to this work. All authors critically read and approved the manuscript.

### Conflict of interest statement

The authors declare that the research was conducted in the absence of any commercial or financial relationships that could be construed as a potential conflict of interest.
